# Pan-cancer analysis of the role of α2C-adrenergic receptor (ADRA2C) in human tumors and validation in glioblastoma multiforme models

**DOI:** 10.7150/jca.98240

**Published:** 2024-09-09

**Authors:** Xiaoxiao Zhang, Huitong Chen, Chenyang Wang, Chan Chen, Liyan Liu, Shuangfa Nie, Xiang Gao, Ning Huang, Junli Chen

**Affiliations:** 1 Department of Pathophysiology, West China School of Basic Medical Sciences & Forensic Medicine, Sichuan University, Chengdu 610041, China.; 2 Department of Gastrointestinal Surgery, the First Affiliated Hospital of Hebei North University, Zhangjiakou 075061, China.; 3 Department of Neurosurgery and Institute of Neurosurgery, State Key Laboratory of Biotherapy and Cancer Center, West China Hospital, West China Medical School, Sichuan University and Collaborative Innovation Center for Biotherapy, Chengdu 610041, China.; 4 NHC Key Laboratory of Chronobiology (Sichuan University), Chengdu 610041, China.

**Keywords:** α2C-adrenergic receptor, pan-cancer analysis, prognosis, apoptosis, invasion, glioblastoma multiforme

## Abstract

**Background:** Several studies have reported the relationship between α2C-adrenergic receptor (ADRA2C) and both neoplastic and non-neoplastic diseases. However, a comprehensive pan-cancer analysis is currently lacking.

**Methods:** Utilizing the RNA sequencing (RNA-seq) datasets from The Cancer Genome Atlas (TCGA) database, the roles of ADRA2C in human pan-cancer were analyzed through a variety of bioinformatics approaches, including R programming language and single-cell sequencing data analysis,* et al*. Besides, cell migration assay and immunochemistry were employed to further validate the role of ADRA2C in glioblastoma multiforme (GBM) cell lines and GBM mouse model.

**Results:** A total of 33 cancer types were involved in this study. It was revealed that the expression level of ADRA2C varied across different clinical stages in patients with breast invasive carcinoma (BRCA), esophageal adenocarcinoma (ESCA), kidney renal papillary cell carcinoma (KIRP) and lung squamous cell carcinoma (LUSC). Meanwhile, it was found that ADRA2C may play roles in prognosis of adrenocortical carcinoma (ACC), glioblastoma multiforme and lower grade glioma (GBM-LGG), and uveal melanoma (UVM). Functional enrichment analysis suggested that ADRA2C expression level was highly correlated with neuronal system-related pathways. Moreover, ADRA2C may be a promising diagnostic marker for cervical squamous cell carcinoma and endocervical adenocarcinoma (CESC), cholangiocarcinoma (CHOL), GBM, GBMLGG, kidney chromophobe (KICH), and KIRP. Additionally, ADRA2C expression level was correlated with the levels of several infiltrating cells and immune checkpoint genes. Besides, the single-cell sequencing data analysis indicated that ADRA2C played a role in multiple tumor development processes in GBM, retinoblastoma (RB), and UVM. Finally, *in vitro* and *in vivo* experiments confirmed that the expression level of ADRA2C may be associated with glioma cell migration, apoptosis, and invasion.

**Conclusion:** ADRA2C exhibited to play a notable role in several cancer types, suggesting that ADRA2C could serve as a promising biomarker or target for cancer diagnosis, prognosis, and treatment, particularly for GBM.

## Introduction

Cancer poses a notable global threat to both human health and economic development. Studies have primarily concentrated on identifying key pathogenic factors, understanding their mechanisms, discovering reliable biomarkers for diagnosis and clinical prognosis, and developing new therapeutic interventions 1-3. An increasing number of studies have confirmed that genetic markers could provide new insights into the aforementioned issues 4-6.

According to the GeneCards database (https://www.genecards.org/), α2C-adrenergic receptor (ADRA2C) is a subtype of G protein-coupled receptors (GPCRs), which is located in 4p16.3 genomic region. ADRA2C plays a crucial role in regulating neurotransmitter release from sympathetic nerves and adrenergic neurons in the central nervous system, which is involved in disorders, such as schizophrenia 7, heart failure 8, and renal failure 9. Nevertheless, prior research demonstrated that ADRA2C is involved in the tumorigenesis of diverse cancer types, such as breast cancer 10 and colorectal cancer 11, suggesting that ADRA2C may be a novel biomarker for the diagnosis or treatment of cancer. However, no study has reported the correlation between ADRA2C and pan-cancer, and the function of ADRA2C in pan-cancer remains elusive.

To assess the role of ADRA2C in pan-cancer development, a comprehensive bioinformatics analysis was conducted to explore the relationship between ADRA2C and pan-cancer using The Cancer Genome Atlas (TCGA), UCLAN, Gene Expression Profiling Interactive Analysis (GEPIA), cBioPortal, and CancerSEA databases. Additionally, the results of the bioinformatics analysis were validated *in vivo* and *in vitro* through cell migration assays, Western blotting, and immunohistochemistry (IHC).

## Methods

### ADRA2C expression level in pan-cancer

The ADRA2C gene expression TPM data were obtained from UCSC XENA (https://xenabrowser.net/datapages/), including RNAseq data from TCGA and GTEx databases. The data were processed through Toil 12. For paired tumor and normal tissues in TCGA pan-cancer, the RNA-seq datasets were downloaded from TCGA level 3 and converted from FPKM (Fragments Per Kilobase per Million) format into TPM (transcripts per million reads) format. Meanwhile, the data were log2 transformed before analysis 13, 14. The data were analyzed using R packages (Version 3.6.3), including ggplot2 (Version 3.3.3) and ggradar (Version 0.2) packages.

### ADRA2C expression across different clinical stages in pan-cancer

For ADRA2C expression analysis across different clinical stages, the data were downloaded from GEPIA database (http://gepia2.cancer-pku.cn/) based on TCGA database. The log2(TPM+1) was utilized for log-scale transformation. Differential gene expression analysis was conducted through one-way analysis of variance (ANOVA), using pathological stage as a variable for calculating differential expression.

### Survival analysis

Kaplan-Meier (KM) analysis was performed to explore the correlation between ADRA2C expression and prognosis of patients with various human cancer types. The RNA-seq data were processed as described above 14. Three clinical outcomes were evaluated, including overall survival (OS), disease-specific survival (DSS), and progression-free interval (PFI). The data were analyzed using R packages (Version 3.6.3), involving survminer (Version 0.4.9) and survival (Version 3.2-10) packages.

### Genetic alteration analysis of ADRA2C in pan-cancer

The cBioPortal database (https://www.cbioportal.org/) was utilized to collect alteration frequency, structural variant data, mutation type, mutated site information, copy number alteration (CNA), and three-dimensional (3D) structure of the protein across all TCGA tumors. In addition, promoter DNA methylation levels of ADRA2C in pan-cancer were obtained based on TCGA database from the UCLAN database (http://ualcan.path.uab.edu/tutorial.html). The beta-value indicates the level of DNA methylation, ranging from 0 (unmethylated) to 1 (fully methylated). Different beta cut-off values have been considered to indicate hypermethylation [beta-value: 0.7-0.5] or hypomethylation [beta-value: 0.3-0.25].

### Gene set enrichment analysis (GSEA) of ADRA2C

Gene set enrichment analysis (GSEA) was performed using gene set collections from the MSigDB 15. Data processing involved R packages, such as DESeq2 (Version 1.26.0) for differential gene expression analysis, cluster Profiler (Version 3.14.3) for functional annotation 16, and ggplot2 (Version 2.3.3) for visualization. Enrichment with false discovery rate (FDR)<0.25 and adjusted *p*-value<0.05 was considered as highly confident and statistically significant. STRING database (https://string-db.org/) was utilized to analyze potential protein interactions with ADRA2C. Then the relevant genes obtained were subjected to protein-protein interaction (PPI) analysis. A confidence score > 0.7 was set as the significance threshold. Then Cytoscape software (v3.7.0) was used for visualization and subsequent analysis.

### Receiver operating characteristic (ROC) curve analysis

The TPM-normalized TCGA and GTEx RNAseq data (version 7) were obtained from UCSC XENA (https://xenabrowser.net/datapages/) and processed via the Toil pipeline 12. Subsequently, the data were log2-transformed, and plotting ROC curves and calculation of area under the curve (AUC) values were conducted using pROC (Version 1.17.0.1) and ggplot2 (Version 3.3.3) R packages.

### Immune cell infiltration analysis

The TIMER2 database (http://timer.cistrome.org/) was used to analyze the correlation between ADRA2C expression and immune cell infiltration in pan-cancer. Meanwhile, RNA-seq data were obtained from TCGA database and converted from FPKM format into TPM format. Infiltration levels for various immune cell types were evaluated using the single-sample GSEA (ssGSEA), which was performed through the gsva package (Version 1.34.0) in R (version 3.6.3) 14. Notably, 24 different immune cell types were involved in the analysis 17.

### Correlation of ADRA2C expression with immune checkpoint (ICP) genes

The TISIDB database (http://cis.hku.hk/TISIDB/index.php) was employed to evaluate the correlation between ADRA2C expression and ICP genes.

### Analysis of single-cell sequencing data

The CancerSEA database (http://biocc.hrbmu.edu.cn/CancerSEA/home.jsp) was utilized to explore the correlation between ADRA2C expression and different functional states of various cancer cells at a single-cell level 18. Heatmap was plotted according to the data downloaded from the CancerSEA database using ggplot R package. Moreover, the t-distributed stochastic neighbor embedding (t-SNE) plot was downloaded from the CancerSEA database.

### Cell culture and drug treatment

In this experiment, GL261 and U87 cell lines were cultured in a high-glucose Dulbecco's modified Eagle's medium (DMEM), containing 10% fetal bovine serum (FBS) (ZETA, NY, USA) and minimum essential medium-non-essential amino acids (MEM-NEAA) (Procell Life Science & Technology Co., Ltd., Wuhan, China) supplemented with 15% FBS, respectively. The cells were incubated at 37 °C with 5% carbon dioxide.

For drug treatment, two drugs were utilized in this study: nonselective α2-adrenergic receptor antagonist (phentolamine) and nonselective α2-adrenergic receptor agonist (noradrenaline, NA). The abovementioned drugs were purchased from MedChemExpress Co. Ltd. (Shanghai, China). Cells were divided into three groups as follows: the control group treated with dimethyl sulfoxide (DMSO), the NA group treated with 10μM of NA for GL261 and 2μM for U87, the phentolamine group treated with 10μM of phentolamine for GL261 and 0.005μM for U87.

### An *in vivo* glioblastoma multiforme (GBM) model

A total of 30 healthy C57BL/6J female mice (6-8 weeks, 17-20 g) were purchased from Laboratory Animal Center of Sichuan University (China). Besides, 1 × 10^6^ GL261 cells were injected into right flank subcutaneously. When the average tumor volume was 100 mm^3^, mice were divided into 3 groups and injected with drugs intraperitoneally every other day for 14 days as follows: control group received 100μL of corn oil; NA group received 0.5 mg/kg dissolved in 100μL of corn oil; and phentolamine group received 1 mg/kg dissolved in 100μL of corn oil. When the tumor volume reached 2000 mm^3^, mice were euthanized via cervical dislocation. Tumor tissue samples were subsequently collected, weighed, and fixed in 4% paraformaldehyde for further processing.

### Cell migration assay

The migration ability of GL261 and U87 cells was evaluated by scratch wound healing assay. For this purpose, 6 × 10^5^ GL261 and U87 cells were seeded into 6-well plates. On the following day, a scratch was introduced into the cell layer using a 200μL pipette tip when the cells reached approximately 90% confluence. Subsequently, the cells were twice washed with phosphate-buffered saline (PBS), and subsequently treated separately with DMEM containing DMSO, NA, or phentolamine. The same wounded areas were observed and photographed at different time points using an inverted microscope (Olympus, Tokyo, Japan). ImageJ software was used to measure scratched areas, enabling the calculation of cell migration speed. Percentages cell migration was calculated using the following formula: Cell migration rate = (0 h scratch area - 24h scratch area)/0 h scratch area × 100%.

### IHC

The tumor tissues from C57BL/6J mice were fixed, processed into paraffin sections, and subjected to IHC. Thereafter, the sections were dewaxed and hydrated before antigen retrieval. The primary antibodies (Bax, BCL-2, and MMP2) and a secondary antibody were incubated. The sections were stained with 3,3'-diaminobenzidine (DAB), followed by counterstaining with hematoxylin, dehydration, and sealing. Images were thereafter captured using NanoZoomer2.0 HT.

### Statistical analysis

Shapiro-Wilk test was used to evaluate normal distribution of data. Correlation was assessed using the Pearson correlation coefficient. *p˂*0.05 was considered statistically significant (ns, *p*≥0.05; ∗, *p˂*0.05; ∗∗, *p˂*0.01; ∗∗∗, *p˂*0.001).

## Results

### ADRA2C expression in pan-cancer

**Figure 1:**
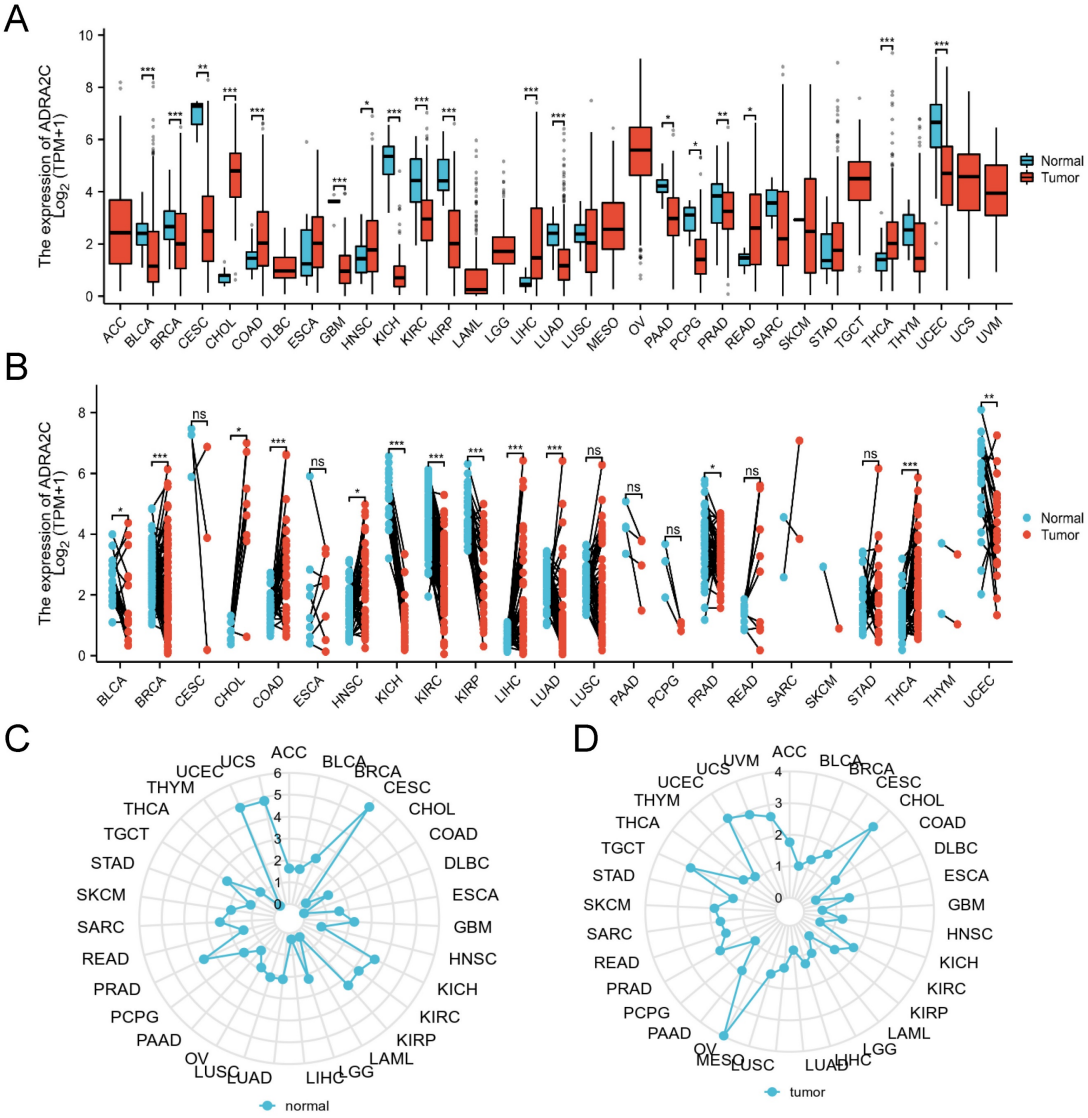
** (A)** Expression of ADRA2C in cancer and normal tissues from TCGA and GTEx databases, analyzed by Mann-Whitney U test. ns, *p*≥0.05; ∗, *p˂*0.05; ∗∗,* p˂*0.01; ∗∗∗, *p˂*0.001. (ACC, adrenalcortical carcinoma; BLCA, bladder urothelial carcinoma; BRCA, breast invasive carcinoma; CHOL, cholangiocarcinoma; COAD, colon adenocarcinoma; DLBC, difuse large B-cell lymphoma; ESCA, esophageal carcinoma; GBM, glioblastoma multiforme; HNSC, Head and Neck squamous cell carcinoma; KICH, kidney chromophobe; KIRC, kidney renal clear cell carcinoma; KIRP, kidney renal papillary cell carcinoma; LAML, acute myeloid leukemia; LGG, brain lower grade glioma; LIHC, liver hepatocellular carcinoma; LUAD, lung adenocarcinoma; LUSC, lung squamous cell carcinoma; MESO, mesothelioma; OV, ovarian serous cystadenocarcinoma; PAAD, pancreatic adenocarcinoma; PCPG, pheochromocytoma and paraganglioma; PRAD, prostate adenocarcinoma; READ, rectum adenocarcinoma; SARC, sarcoma; SKCM, skin cutaneous melanoma; STAD, stomach adenocarcinoma; TGCT, testicular germ cell tumor; THCA, thyroid carcinoma; THYM, thymoma; UCEC, uterine corpus endometrial carcinoma; UVM, uveal melanoma). **(B)** ADRA2C mRNA expression level of paired tumor and normal samples from TCGA. **(C)** mRNA expression of ADRA2C in normal tissues from GTEx database.** (D)** mRNA expression of ADRA2C in tumor tissues from TCGA database.

The expression level of ADRA2C in pan-cancer involving 33 cancer types was analyzed. As illustrated in **Figure 1A**, compared with normal group, ADRA2C mRNA expression level was higher in cholangiocarcinoma (CHOL), colon adenocarcinoma (COAD), head and neck squamous cell carcinoma (HNSC), liver hepatocellular carcinoma (LIHC), rectum adenocarcinoma (READ) and thyroid carcinoma (THCA). Meanwhile, there was a lower ADRA2C mRNA expression level in bladder urothelial carcinoma (BLCA), breast invasive carcinoma (BRCA), cervical squamous cell carcinoma and endocervical adenocarcinoma (CESC), GBM, kidney chromophobe (KICH), kidney renal clear cell carcinoma (KIRC), kidney renal papillary cell carcinoma (KIRP), lung adenocarcinoma (LUAD), pancreatic adenocarcinoma (PAAD), pheochromocytoma and paraganglioma (PCPG), prostate adenocarcinoma (PRAD) and uterine corpus endometrial carcinoma (UCEC).

To further explore the ADRA2C expression level in pan-cancer, the ADRA2C expression level in paired normal and tumor tissues was determined. It was revealed that there was a higher ADRA2C expression level in BLCA, BRCA, CHOL, COAD, HNSC, LIHC, LUAD, and THCA **(Figure 1B)**. Furthermore, ADRA2C expression level was reduced in KICH, KIRC, KIRP, PRAD and UCEC **(Figure 1B)**. Meanwhile, the normal tissue data from GTEx database indicated a higher ADRA2C mRNA expression level in CESC, KICH, KIRC, KIRP, PRAD, UCEC, and UCS **(Figure 1C)**. For tumor tissue data from TCGA, a higher ADRA2C mRNA expression level was found in CHOL, OV, TGCT, and UCEC **(Figure 1D)**.

### The relationship between ADRA2C expression level and clinicopathological stage in pan-cancer

**Figure 2:**
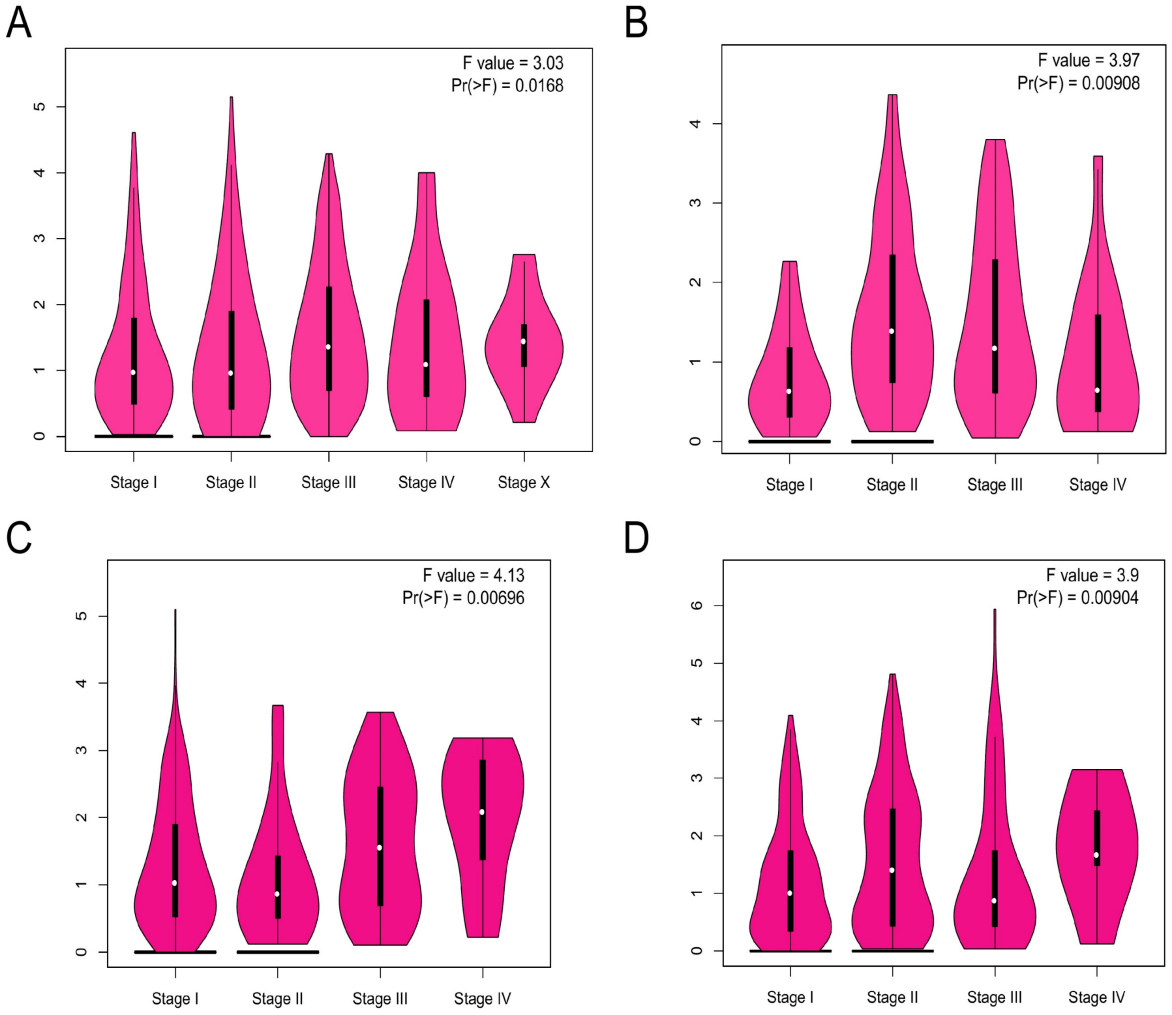
The correlation between ADRA2C expression level and the pathological major stages in BRCA **(A)**, ESCA **(B)**, KIRP **(C)** and LUSC **(D)**. Log2 (TPM+1) was used for log scale.

The ADRA2C expression level in different pathological stages in pan-cancer was analyzed. The results indicated that there was a pathological stage-specific expression level of ADRA2C in BRCA, esophageal adenocarcinoma (ESCA), KIRP, and lung squamous cell carcinoma (LUSC) **(Figures 2A, 2B, 2C** and **2D**, *p*<0.05).

### Survival analysis

**Figure 3:**
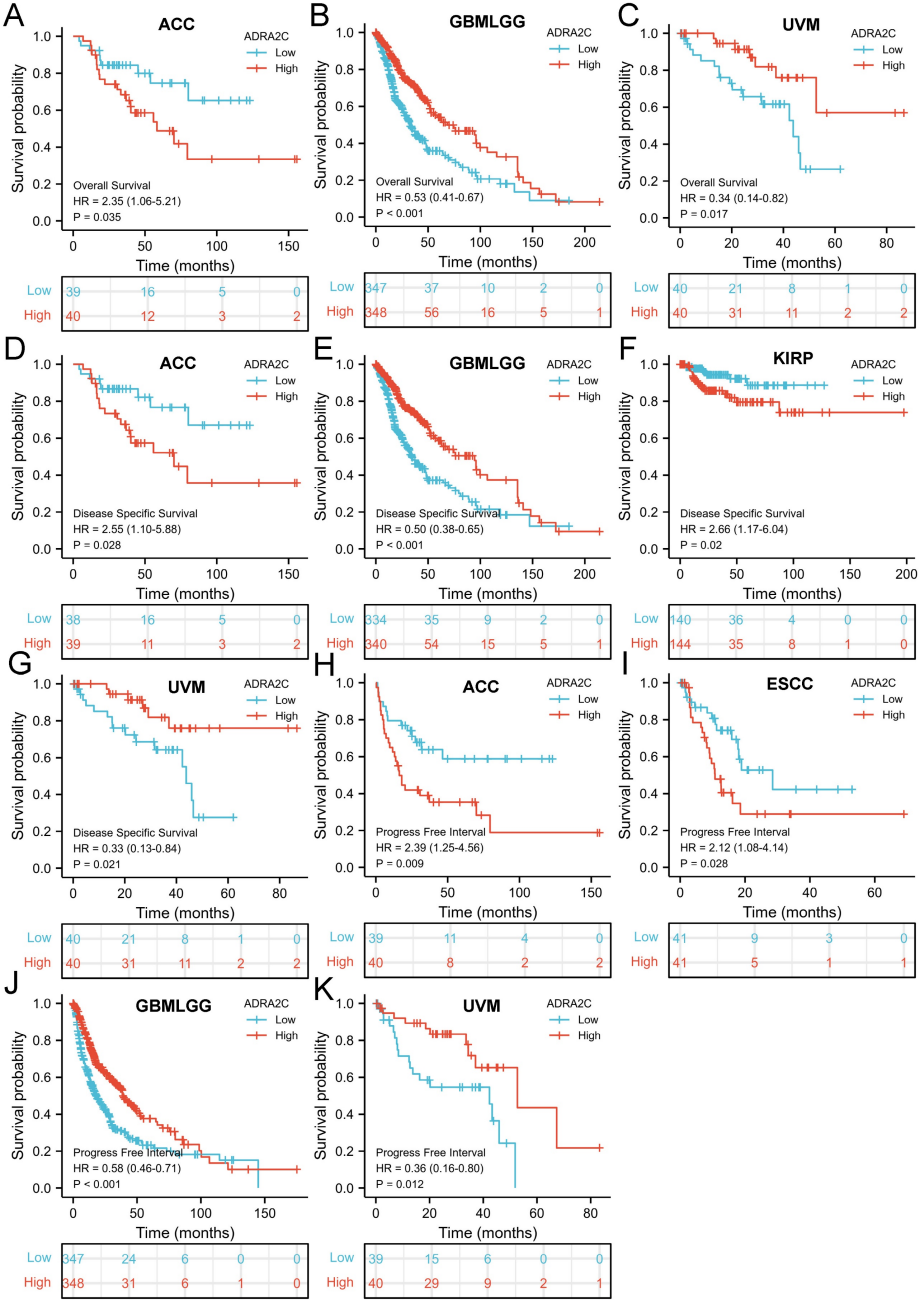
Kaplan-Meier survival curve of human cancers with high and low YTHDF1 expression. Kaplan-Meier survival curves of overall survival for patients stratified by the different expressions of ADRA2C in ACC **(A)**, GBMLGG **(B)** and UVM **(C)**. Kaplan-Meier survival curves of disease specific survival for patients stratified by the different expressions of ADRA2C in ACC **(D)**, GBMLGG **(E)**, KIRP **(F)** and UVM **(G)**. Kaplan-Meier survival curves of progress free interval for patients stratified by the different expressions of ADRA2C in ACC **(H)**, ESCC **(I)**, GBMLGG **(J)**, and UVM **(K)**. Red and blue curves represent patients with high and low expression of ADRA2C, respectively.

The correlation between ADRA2C expression level and prognosis in pan-cancer was evaluated. It was revealed that a high ADRA2C expression level was associated with a favorable OS in GBM-LGG (HR= 0.53, *p*<0.001) and uveal melanoma (UVM) (HR= 0.34, *p*=0.017) **(Figures 3B and 3C)**, while a high ADRA2C expression level was correlated with a poor OS in adrenocortical carcinoma (ACC) (HR=2.35, *p*=0.035) **(Figure 3A)**. In addition, a high ADRA2C expression level was associated with a favorable DSS in GBMLGG (HR=0.50, *p*<0.001) and UVM (HR=0.33, *p*=0.021) **(Figures 3E and 3G)**, while a high ADRA2C expression level was correlated with a poor DSS in ACC (HR=2.55, *p*=0.028) and KIRP (HR=2.66, *p*=0.028) **(Figures 3D** and **3F)**.

Moreover, a high ADRA2C expression level was associated with a favorable PFI in GBMLGG (HR=0.58, *p*<0.001) and UVM (HR=0.36, *p*=0.012) **(Figures 3J** and **3K)**, while a high ADRA2C expression level was correlated with a poor PFI in ACC (HR=2.39, *p*=0.009), and esophageal squamous cell carcinoma (ESCC) (HR=2.12, *p*=0.028) **(Figures 3H** and **3I)**. Finally, there was no relationship between ADRA2C expression level and prognosis of BLCA, BRCA, CESC, CHOL, COAD, DLBC, ESCA, esophageal adenocarcinoma (ESAD), GBM, HNSC, KICH, KIRC, LAML, LGG, LIHC, LUAD, LUSC, mesothelioma (MESO), oral squamous cell carcinoma (OSCC), OV, PAAD, PCPG, PRAD, READ, sarcoma (SARC), SKCM, STAD, TGCT, THCA, THYM, UCEC, and UCS. Taken together, we considered that ADRA2C was closely related with the prognosis of ACC, GBMLGG and UVM.

### Genetic alteration analysis of ADRA2C in pan-cancer

**Figure 4:**
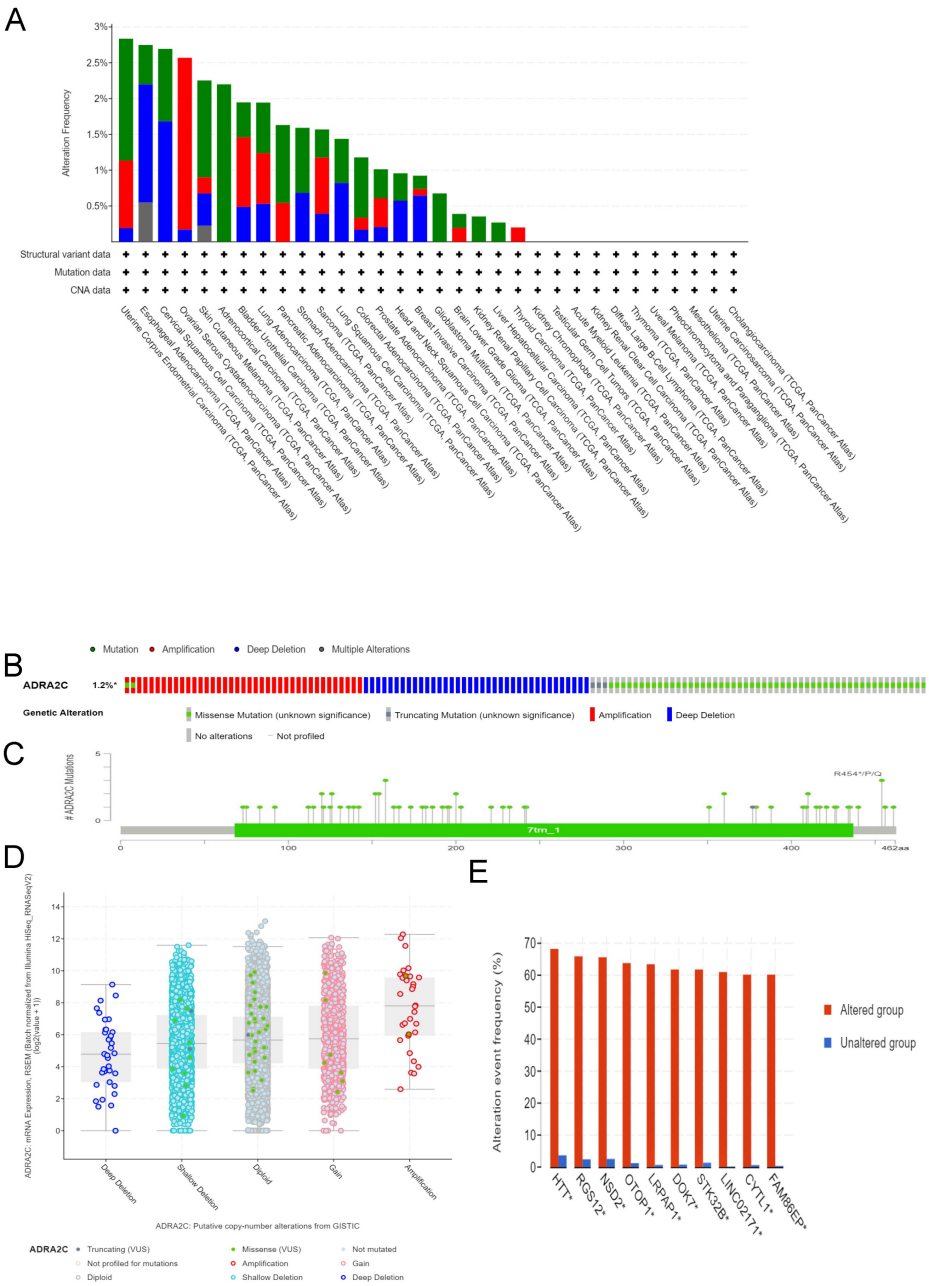
Genetic alteration analysis of ADRA2C in pan-cancer from TCGA database. **(A)** Genetic features of ADRA2C, including mutation, amplification, and deletion, in various cancers of TCGA analyzed by cBioPortal database. **(B)** The total mutations in the ADRA2C gene were evaluated using a genome-wide pan-cancer analysis in the cBioPortal database. **(C)** The ADRA2C genetic alteration frequency with various types of mutations was assessed using the cBioPortal database. **(D)** The genetic alteration types of ADRA2C in pan-cancer. **(E)** The related genes alteration frequency in ADRA2C altered group and unaltered group.

**Figure 5:**
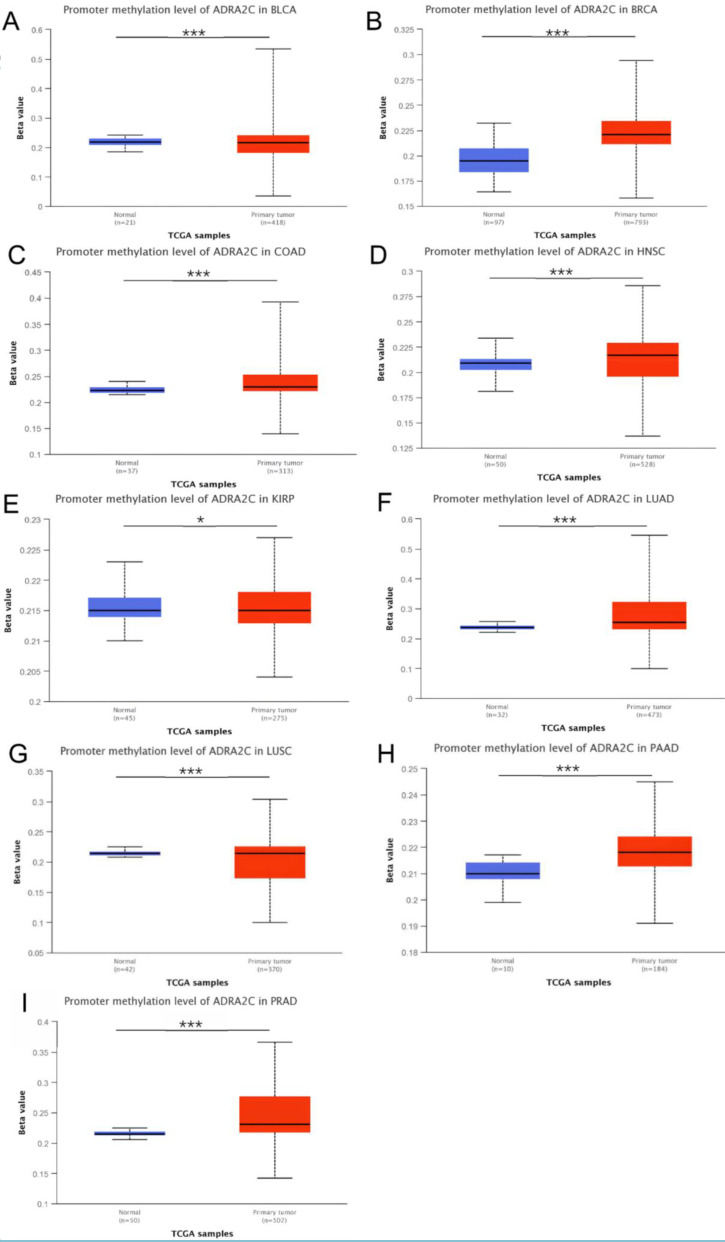
The ADRA2C methylation level analysis in pan-cancer from UALCAN database. ∗*p* < 0:05, ∗∗*p* < 0:01, and ∗∗∗*p* < 0.001.

Different genetic mutations of ADRA2C in pan-cancer samples from cBioPortal database are illustrated in** Figure 4**. As displayed in **Figure 4A**, the frequency of ADRA2C alteration (2.2%) was the highest in adrenocortical carcinoma with 'mutation' as the primary type, and all adrenocortical carcinoma patients had ADRA2C mutation. The 'amplification' type of CNA had the highest incidence (2.4%) in ovarian serous cystadenocarcinoma. Meanwhile, the highest frequency in the 'deep deletion' type of ADRA2C was found in cervical squamous cell carcinoma patients. **Figures 4B and 4C** show mutation types, location sites, and case number of ADRA2C. It was found that missense mutation of ADRA2C was the main type of genetic alteration, which was detected in 60 cases, while truncating mutation was detected in only 3 cases. It was also revealed that the most frequent copy-number alterations of ADRA2C were amplification, gain function, diploid, shallow deletion, and deep deletion **(Figure 4D)**. As displayed in **Figure 4E**, the genetic alterations of HTT, RGS12, NSD2, OTOP1, LRPAP, DOK7, STK32B, LINC02171, CYTL1, and FAM86EP were more frequent in the ADRA2C altered group than those in the ADRA2C unaltered group. For ADRA2C promoter methylation level, it was found that ADRA2C hypomethylation was identified in most tumor types. Compared with normal tissues, there was a significantly higher methylation level in BRCA, COAD, HNSC, LUAD, PAAD, and PRAD** (Figure 5)**.

### Functional enrichment analysis of ADRA2C in pan-cancer

**Figure 6:**
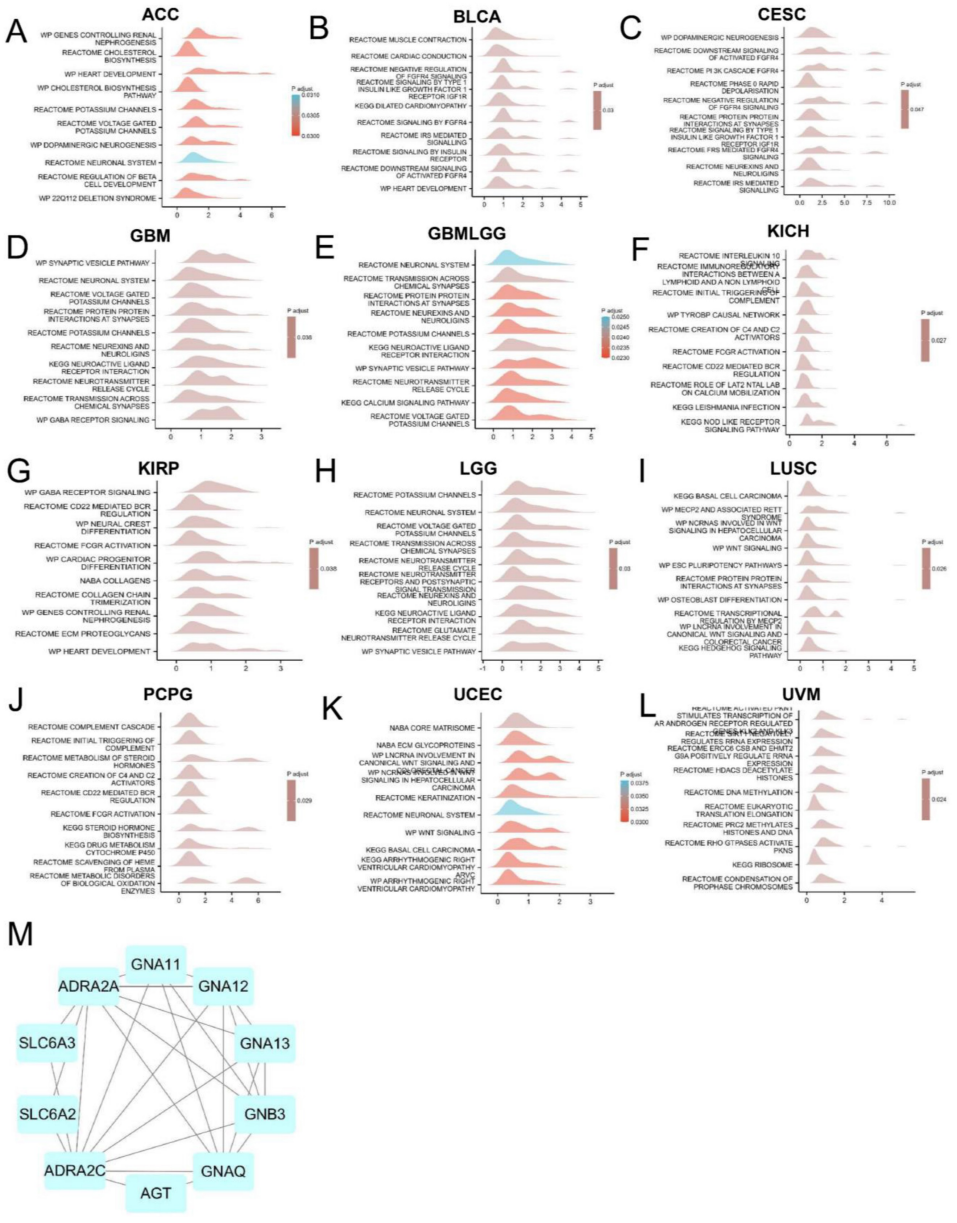
GSEA analysis of ADRA2C.** (A-L)** Top 10 GSEA terms in pan-cancer. **(M)** The binding proteins of ADRA2C were analysed by STRING website and Cytoscape software.

To explore the molecular mechanism of ADRA2C in different tumors, the pathways in which ADRA2C could be involved in pan-cancer were evaluated using GSEA. The results revealed that ADRA2C was commonly correlated with neuronal system-related pathways, especially in ACC, CESC, GBM, GBMLGG, KIRP, and lower grade glioma (LGG) **(Figures 6A, 6C, 6D, 6E, 6G, 6H)**. Furthermore, using a protein-protein interaction (PPI) network, it was indicated that CD161 was closely associated with ADRA2A, AGT, GNAT11, GNA12, GNA13, GNAQ, GNB3, SLC6A2 and SLC6A3 proteins **(Figure 6M)**.

### ROC curve analysis

**Figure 7:**
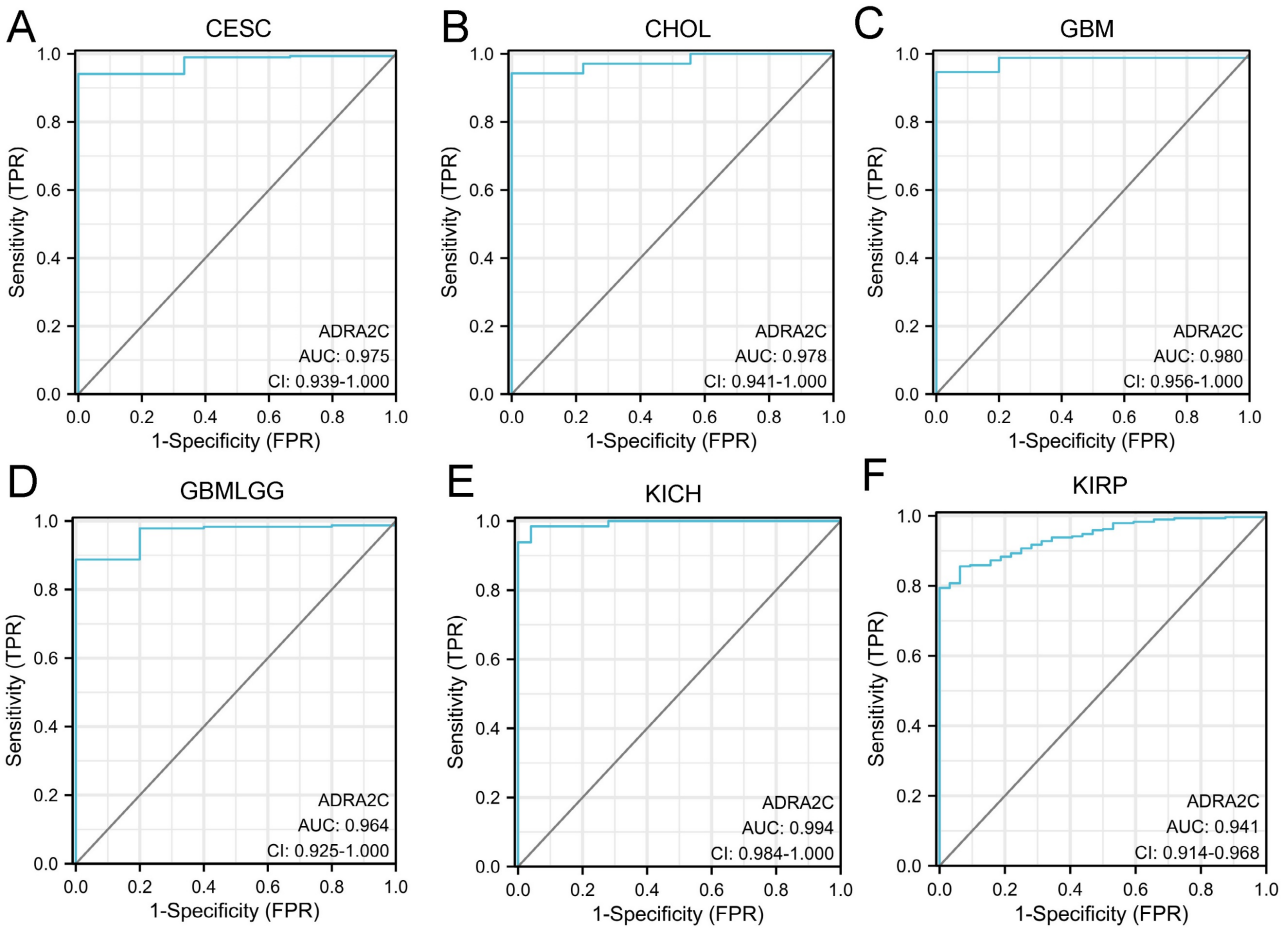
ROC analysis of ADRA2C in pan-cancer. ROC curves showed ADRA2C has a high accuracy for diagnosis of CESC **(A)**, CHOL **(B)**, GBM **(C)**, GBMLGG** (D)**, KICH **(E)** and KIRP **(F)**.

The diagnostic ability of ADRA2C in pan-cancer was evaluated through ROC curve analysis. It was revealed that the AUC value in CESC, CHOL, GBM, GBMLGG, KICH, and KIRP was higher than 0.9 **(Figure 7)**, suggesting that ADRA2C could be a notable diagnostic mark**e**r for these tumors.

### Immune cell infiltration analysis

In order to fully explore the correlation between ADRA2C expression and immune cell infiltration in various cancer types, immune cell infiltration analysis was conducted utilizing two sources.

**Figure 8:**
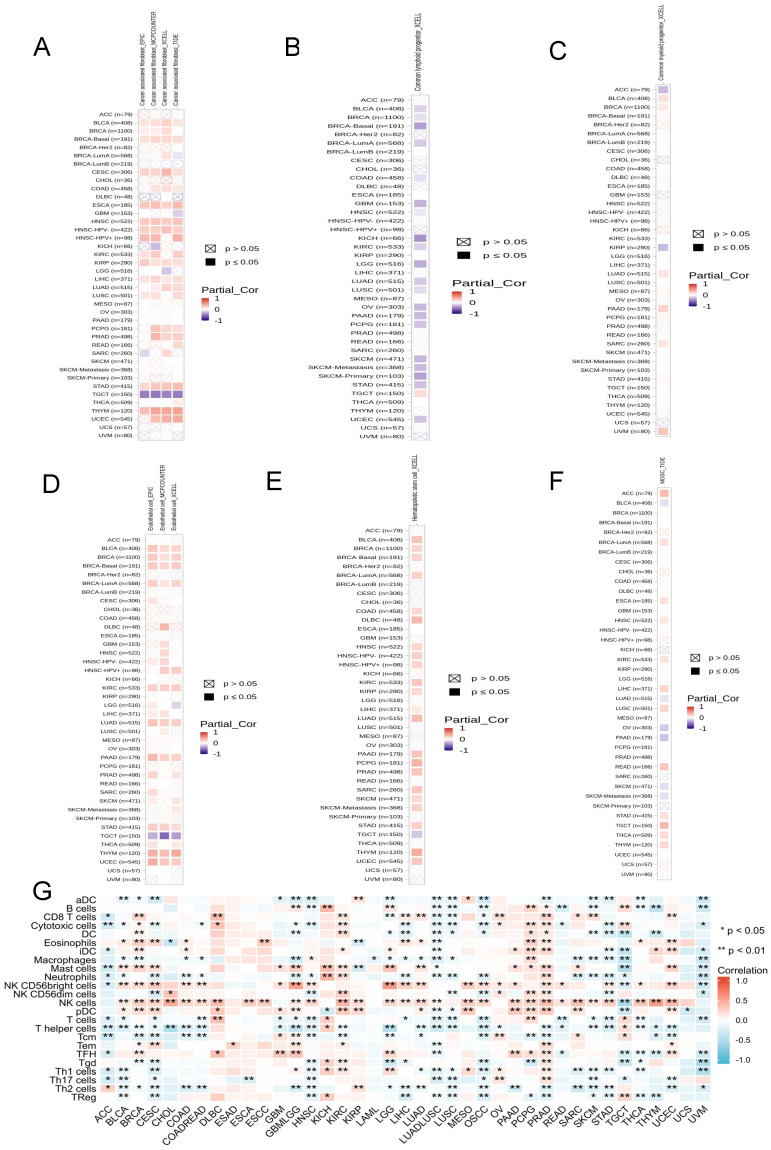
The relationship between ADRA2C expression and immune infiltration in pan-cancer. **(A-F)** represents cancer-associated fibroblast, common lymphoid progenitor, common myeloid progenitor, endothelial cell, hematopoietic stem cell and myeloid derived suppressor cells in TIMER2.0 database, respectively. **(G)** represents the correlation between ADRA2C expression and 24 immune cells infiltration in pan-cancer from published works.

The data from TIMER 2.0 indicated that there was a positive correlation between ADRA2C expression level and cancer-associated fibroblast in BLCA, BRCA-Basal, CESC, ESCA, HNSC, KIRP, LIHC, STAD, and THYM, as well as a negative correlation in TGCT **(Figure 8A)**. For the common lymphoid progenitor, a positive correlation was found in TGCT, while a negative correlation was identified in BLCA, BRCA-Basal, COAD, GBM, HNSC, KICH, KIRC, LGG, LUAD, LUSC, OV, PAAD, PCPG, skin cutaneous melanoma (SKCM), STAD, and UCEC **(Figure 8B)**.

There was a positive correlation between ADRA2C expression level and the common myeloid progenitor in BLCA, BRCA, LUAD, PAAD, SARC, and UVM, while a negative correlation was found in ACC and KIRP **(Figure 8C)**. For the endothelial cells, there was a significantly positive correlation in BLCA, BRCA, BRCA-Basal, BRCA-lumA, KIRC, LUAD, PAAD, STAD, THYM, and UCEC, whereas there was a negative correlation in TGCT **(Figure 8D)**. In hematopoietic stem cells, there was a positive correlation in BLCA, BRCA, BRCA-Basal, BRCA-lumA, COAD, DLBC, HNSC, KIRC, KIRP, LIHC, LUAD, PAAD, PCPG, PRAD, SARC, SKCM, STAD, THYM, and UCEC, while a negative correlation was identified in TGCT **(Figure 8E)**.

Regarding myeloid-derived suppressor cells, there was a positive correlation in ACC, BRCA-lumA, ESCA, HNSC, KIRC, LUSC, READ, STAD, TGCT, THCA, and THYM, while a negative correlation was identified in BLCA, LUAD, OV, PAAD, SKCM, and SKCM-metastasis **(Figure 8F)**. Previous studies showed that there was a significantly positive correlation between ADRA2C expression level and most of the immune infiltration cell types in ACC, KICH, KIRC, OV, PCPG, and PRAD, and there was a significantly negative correlation between ADRA2C expression level and most of the immune infiltration cell types in LUAD-LUSC, LUSC, OSCC, STAD, and UVM **(Figure 8G)**. It is noteworthy that the data from TIMER2.0 and TCGA exhibited mostly consistent results for B cells, CD8 T cells, dendritic cells (DCs), eosinophils, macrophages, mast cells, neutrophils, natural killer (NK) cells, T follicular helper (TFH) cells, and regulatory T (Treg) cells (data were not shown).

Taken together, all these clues demonstrated ADRA2C expression was associated with several immune cell infiltration and thus regulated the tumor microenvironment.

### Correlation of ADRA2C expression level with immunomodulatory genes

**Figure 9:**
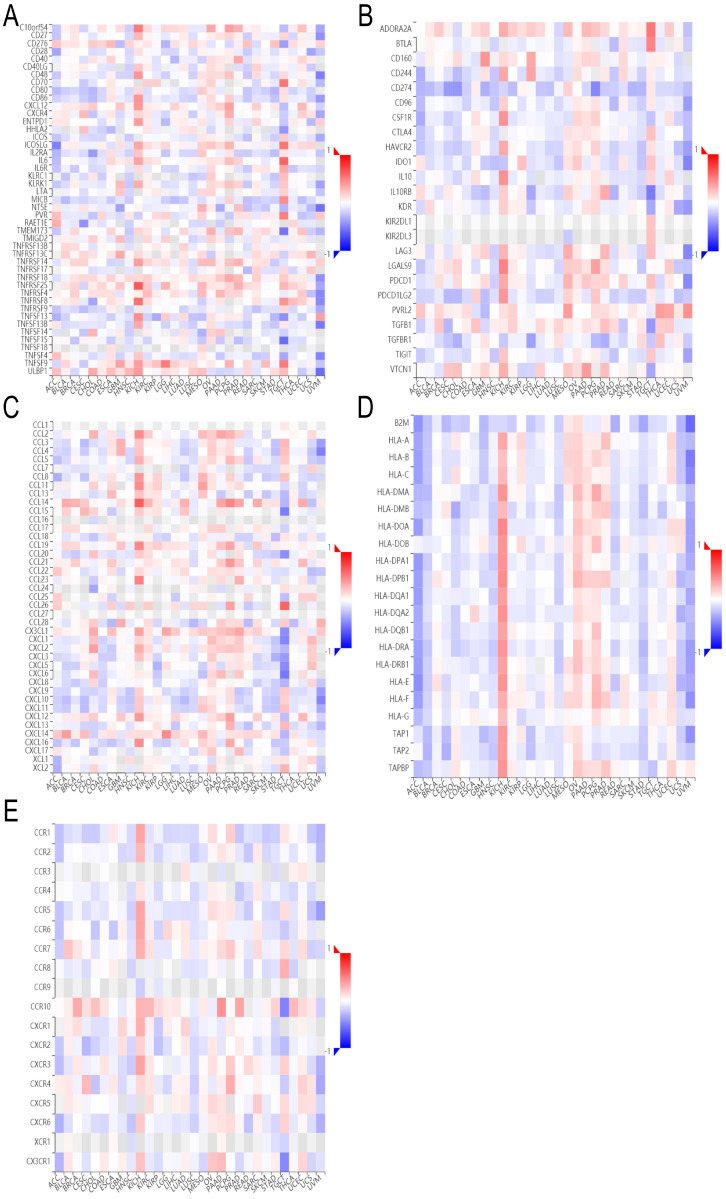
The heatmap of correlation between ADRA2C expression and immunomodulatory genes.** (A-E)** represent immunostimulators, immunoinhibitors, chemokines, MHC molecules and receptors, respectively.

It was found that ADRA2C expression level was positively correlated with all immunostimulators in OV **(Figure 9A)**, while it exhibited a negative correlation with nearly all immunoinhibitors in UVM **(Figure 9B)**. Regarding major histocompatibility complex (MHC) molecules, there was a positive correlation between ADRA2C expression level and KICH, as well as OV, while there was a negative correlation between ADRA2C expression level and ACC, BLCA, COAD, HNSC, LGG, LUSC, SARC, SKCM, STAD, and UVM **(Figure 9D)**. These data indicated that ADRA2C expression was related to several immunomodulatory genes associated with tumor gene therapies.

### Analysis of single-cell sequencing data

**Figure 10:**
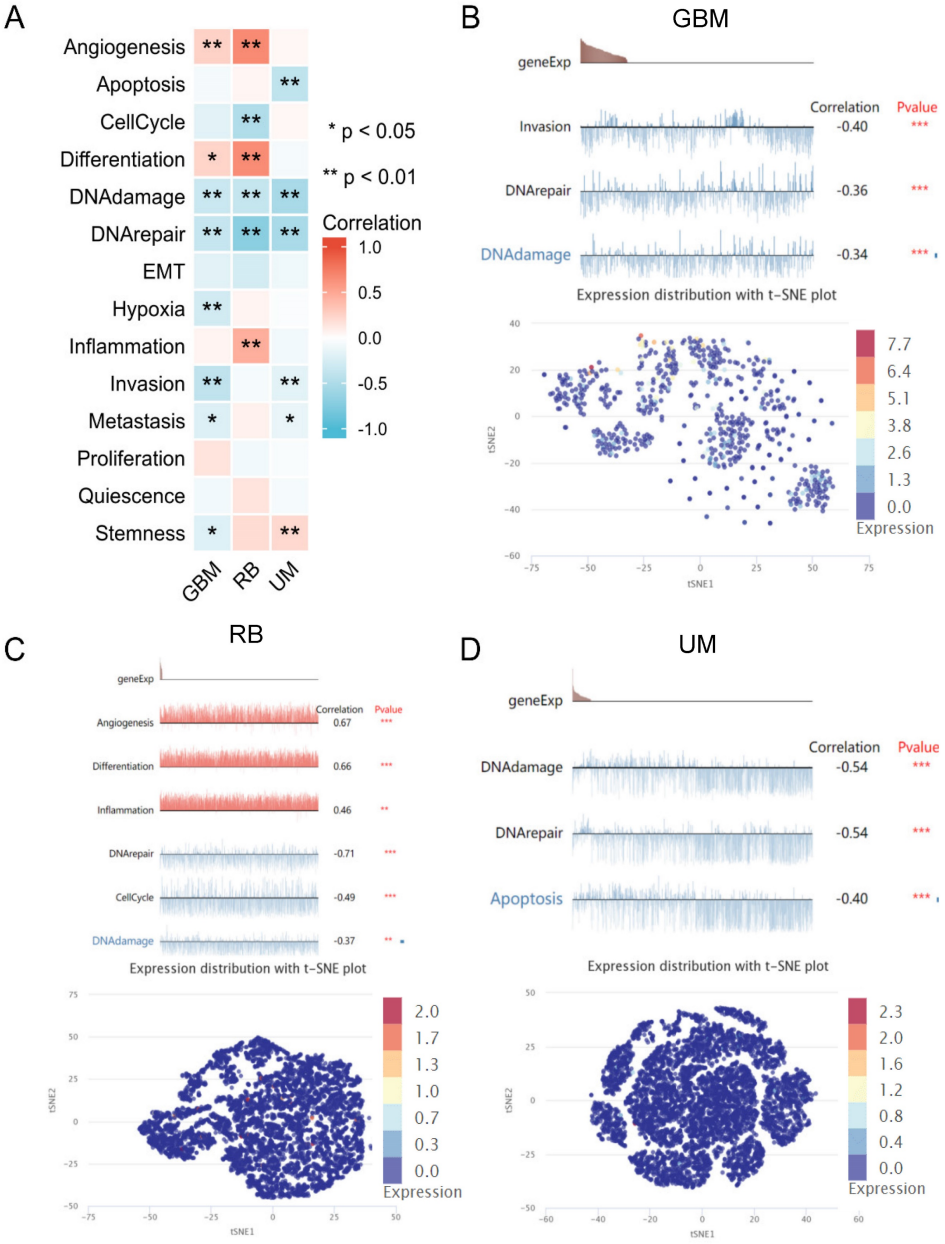
The relationship between ADRA2C expression levels and different functional states at single-cell levels in various cancers. **(A)** showed the correlation between ADRA2C expression and different functional states in pan-cancer. **(B, C, D)** presented the correlation between ADRA2C expression and different functional states as well as ADRA2C expression profiles at single cells from GBM, RB and UM by T-SNE diagram, respectively.

The analysis of single-cell sequencing data indicated that ADRA2C expression level was significantly positively correlated with angiogenesis and differentiation in GBM and retinoblastoma (RB), as well as inflammation in RB and stemness in UM **(Figure 10)**. However, it was significantly negatively correlated with DNA damage and DNA repair in GBM, RB, and uveal melanoma (UM), as well as cell invasion and metastasis in GBM and UM. Notably, ADRA2C expression level was significantly positively associated with stemness in UM, while negatively with stemness in GBM. Meanwhile, ADRA2C expression level was significantly positively correlated with inflammation in RB, while ADRA2C expression level was significantly negatively associated with apoptosis in UM and cell cycle in RB **(Figure 10A)**. Furthermore, the T-SNE diagram illustrates the ADRA2C expression level in single cells of GBM, RB, and UVM **(Figures 10B, 10C, and 10D)**. For GBM, ADRA2C expression level ranged from 0 to 7.689; for RB, the range was from 0 to 2.005; and for UVM, it was from 0 to 2.346. These findings suggested that ADRA2C could play a role in the progression of GBM, RB, and UVM.

### ADRA2C expression level was correlated with migration of glioma cell lines

**Figure 11:**
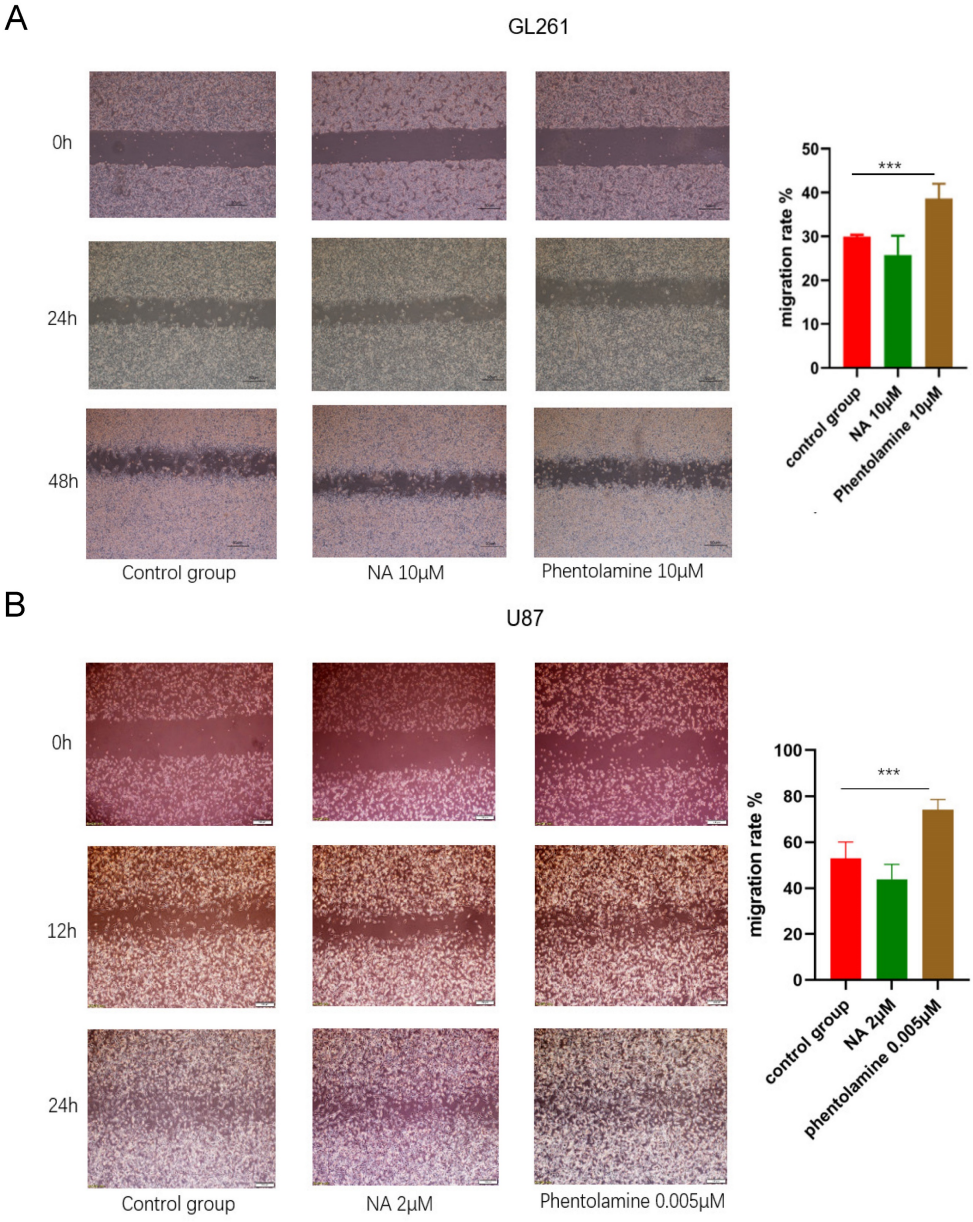
Effect of ADRA2C on the migration of GL261 cells **(A)** and U87 cells** (B)**. Representative images of GL261 cells and U87 cells treated with serum-free Dulbecco's Minimal Essential Medium (DMEM)/high glucose/Penicillin-/Streptomycin (Pen/Strep), NA and phentolamine at different times after a scratch was introduced in the monolayer with a 200μL pipette tip. Meanwhile, percentages cell migration was calculated using the following formula: Cell migration rate = (0 h scratch area - 24h scratch area)/0 h scratch area × 100%. Scale bar, 50 µm. The data are presented as mean ± standard deviation. (*n* = 3, ***p < 0.001, two-way ANOVA).

The wound healing assay was performed to explore the role of ADRA2C in glioma cell migration. Compared with the control group, in both GL261 and U87 cells, the migration speed increased in the group treated with the ADRA2C antagonist, phentolamine, while it decreased in the group treated with the ADRA2C agonist, norepinephrine **(Figure 11)**, suggesting that a lower ADRA2C expression level may promote the GBM cell migration.

### ADRA2C could play roles in glioma cell apoptosis and invasion

**Figure 12:**
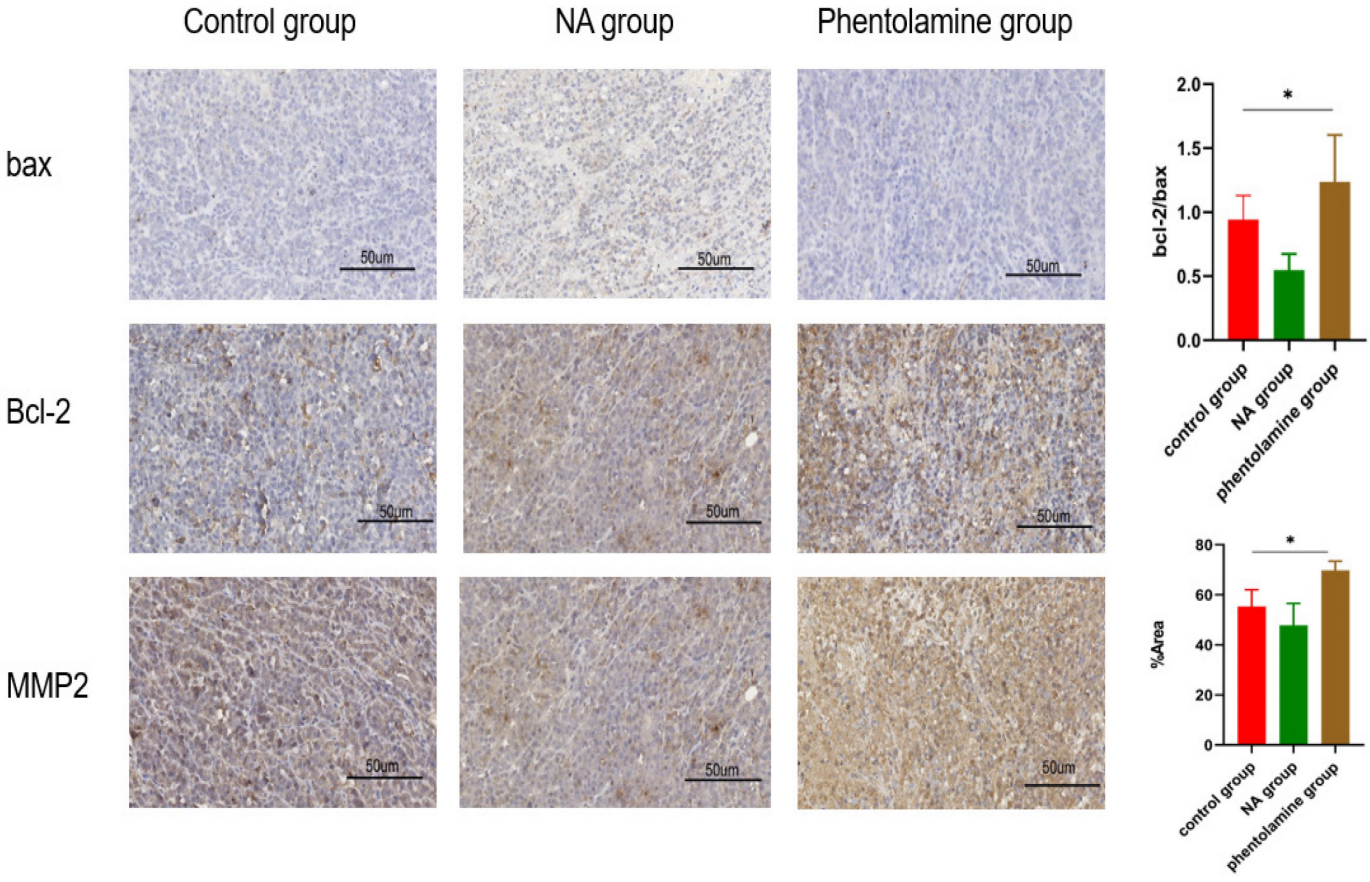
Phentolamine promotes glioma apoptosis and invasion and NA reduces glioma apoptosis and invasion. GL261 cells were injected subcutaneously into the right flanks of mice to establish tumors. When tumors reached approximately 100 mm^3^, mice received an intraperitoneal injection of corn oil (control group), NA (0.5 mg/kg) or phentolamine (1 mg/kg) every other day for 14 days, separately. Immunohistochemistry analysis was utilized to assess the expression the apoptosis-related proteins (bax and bcl-2) and invasion-related protein (MMP2) in tumor tissues of mouse glioma model. Pictures are shown at 40× magnification. Scale bar, 50 µm. The data are presented as mean ± standard deviation. (n = 3, *p < 0.05, two-way ANOVA).

To further evaluate whether ADRA2C could be involved in the process of glioma tumorigenesis, the expression levels of Bax, Bcl-2, and MMP2 in tumor tissues of mouse glioma models were detected using IHC. The results indicated that compared with control group, Bcl2/Bax ratio and MMP2 expression level were elevated in phentolamine-treated group, while decreased in norepinephrine-treated group (**Figure 12**). This suggested that a lower ADRA2C expression level may promote the glioma cell apoptosis and invasion.

## Discussion

Due to high mortality rates of diverse cancer types, the lack of effective biomarkers for diagnosis, prognosis, and treatment should be urgently eliminated. ADRA2C plays a notable role in modulating neurotransmitter release from sympathetic nerves and adrenergic neurons in the central nervous system. Recent research highlights its involvement in non-neoplastic conditions, such as schizophrenia 7, attention deficit hyperactivity disorder (ADHD), and heart failure. However, limited studies have explored its association with cancer development, including breast cancer 10, 17, glioma 20, and colorectal cancer 21. There is a scarcity of systematic analyses elucidating the role of ADRA2C across different types of cancer. Pan-cancer analysis could be a comprehensive method to investigate the role of ADRA2C in various human cancer types.

Utilizing the TCGA data, GTEx RNA-seq data, and paired tumor and normal tissue data altogether, this pan-cancer analysis revealed that the mRNA expression level was higher in CHOL, COAD, HNSC, LIHC, READ, and THCA. Meanwhile, there was a lower ADRA2C mRNA expression level in BLCA, BRCA, CESC, GBM, KICH, KIRC, KIRP, LUAD, PRAD, and UCEC. These results were mainly consistent with those reported previously 10, 21, as well as data from other databases, such as The Human Protein Atlas (HPA) database (https://www.proteinatlas.org/). KM survival analysis confirmed that a high ADRA2C mRNA level was associated with a favorable prognosis in GBMLGG and UVM, while a high ADRA2C expression level was correlated with a poor prognosis in ACC, suggesting that ADRA2C may be a protective factor in GBMLGG and UVM patients, as well as being a risk factor for ACC. Remarkably, there was no relationship between ADRA2C expression level and the prognosis of breast cancer, which was inconsistent with previous studies 10, 22. This phenomenon may have multifaceted causes, including variations in sample size, the characteristics of samples tested, and the methods of detection. Alternatively, ADRA2C emerged to be capable of discerning cancer patients from healthy subjects with notable sensitivity and specificity in CESC, CHOL, GBM, GBMLGG, KICH, KIRP.

Recently, gene mutation-based therapy has become a significant concern in the field of cancer treatment 23, 24. Previous studies have discovered that global DNA hypomethylation could be recognized as a common hallmark of tumor 25, 26. Moreover, alterations in DNA methylation level in cancer have been considered as a promising diagnostic, prognostic, predictive and treatment biomarker 27. According to the analysis of genetic alterations performed in the present study, different ADRA2C mutation types were included and alterations in DNA methylation level appeared in several cancer types, demonstrating that ADRA2C may participate in the tumorigenesis, especially in BLCA, BRCA, COAD, HNSC, KIRP, LUAD, LUSC, PAAD, and PRAD.

According to the GSEA and PPI network analysis of ADRA2C, it was found that it was mainly correlated with neuronal system-related pathways and several GPCR pathway-related proteins. Previous studies have demonstrated that the ADRA2C related proteins were associated with diver cancer types, such as breast cancer 28, 29, acute myeloid leukemia 30, lung cancer 31, and hepatocellular carcinoma 32. This suggests that ADRA2C may play a role in several processes of tumorigenesis. Thus, single-cell sequencing was conducted in the present study, and it was found that ADRA2C could play a role in angiogenesis, differentiation, DNA damage and repair, invasion, and metastasis in GBM, RB, and UM. To validate the prediction results by mentioned above, we constructed cell and mouse GBM model to investigate the roles of ADRA2C in cell migration, apoptosis, and invasion. According to KEGG database and previous findings, wound healing assay was applied to assess the key apoptosis-related markers, including Bax and Bcl-2, and invasion-related protein, MMP2, for validation the anti-cancer effect and mechanism of ADRA2C drugs in glioma 33-36. It was demonstrated that a lower ADRA2C expression level could accelerate migration, apoptosis, and invasion, further promoting worse prognosis. This conclusion is consistent with that of the bioinformatics analysis as described above.

Over the last decade, researchers have confirmed that the tumor microenvironment (TME) is essential to tumor initiation, progression, metastasis, and immunotherapy 37. On the one hand, infiltration of immune cells is crucial to tumorigenesis, prognosis, drug discovery, and the development of therapeutic strategies for tumors 38-40. The present study revealed that ADRA2C was closely associated with infiltration of multiple immune cells, such as B cells, CD4^+^ T cells, CD8^+^ T cells, NK cells, cytotoxic cells, etc. These findings may provide new insights for the application of ADRA2C in cancer immunotherapy, especially in ACC, BLCA, BRCA, OV, PAAD, STAD, TGCT, THYM, UVM, etc. On the other hand, investigations into immunoregulatory genes, such as *CTLA-4, PD-1, PD-L1*, and MHC molecules may provide novel insights into the discovery of immunotherapeutic agents for combating tumors 41. The application of immune checkpoint inhibitors (ICI) has exhibited a notable efficacy in numerous cancer types, such as colon cancer, gastric cancer, non-small cell lung cancer, and clear cell renal carcinoma 42. Hence, ADRA2C exhibits correlations with various immunostimulators, immunoinhibitors, MHC molecules, cytokines, and receptors. These correlations suggest that ADRA2C might serve as a co-factor in the function of immune checkpoint agents in tumor immunotherapy, especially in ACC, BLCA, COAD, HNSC, KICH, LGG, LUSC, OV, SARC, SKCM, STAD, and UVM. Besides, GPCRs represent the largest class of drug targets currently on the market 43, 44. Consequently, ADRA2C may be a promising target for cancer immunotherapy.

There are some limitations of this study. Firstly, the majority of data were obtained from databases that undergo continuous updates, thereby imposing limitations on the conclusiveness of the findings. Secondly, validation of the role of ADRA2C in the GBM model was primarily based on preliminary experiments. Further research is necessary to authenticate the precision of the outcomes of bioinformatics analysis and to explore the molecular mechanisms involving ADRA2C in GBM and other cancer types. Lastly, the clinical applications of ADRA2C-related products remain elusive and warrant confirmation through more comprehensive *in vivo* and *in vitro* experiments.

## Conclusions

In conclusion, the role of ADRA2C in pan-cancer was systematically evaluated through multiple bioinformatics methods and preliminary experiments. Lower ADRA2C expression level is correlated with GBM patients' poor prognosis. ADRA2C is involved in various processes of tumorigenesis and could serve as a notable target for cancer diagnosis and immunotherapy.

## Abbreviations

ADRA2C: α2C-adrenergic receptor
TCGA: The Cancer Genome Atlas
BRCA: breast invasive carcinoma
ESCA: esophageal adenocarcinoma
KIRP: kidney renal papillary cell carcinoma
LUSC: lung squamous cell carcinoma
ACC: adrenocortical carcinoma
ESCC: esophageal squamous cell carcinoma
GBM: glioblastoma multiforme
GBM-LGG: glioblastoma multiforme and lower grade glioma
THYM: thymoma
UVM: uveal melanoma
RB: retinoblastoma
GPCRs: G protein-coupled receptors
AUC: Area Under the Curve
ROC: Receiver Operating Characteristic
GBM: Glioblastoma Multiforme
LGG: Low-Grade Glioma
OS: overall survival
DSS: disease-specific survival
PFI: progression-free interval
IHC: Immunohistochemistry
CNA: copy number alteration
GSEA: Gene set enrichment analysis
NA: noradrenaline
CHOL: cholangiocarcinoma
DLBC: diffuse large B-cell lymphoma
HNSC: head and neck squamous cell carcinoma
LIHC: liver hepatocellular carcinoma
OV: ovarian carcinoma
PAAD: pancreatic adenocarcinoma
STAD: stomach adenocarcinoma
TGCT: testicular germ cell tumors
THCA: thyroid carcinoma
THYM: thymoma
BLCA: bladder urothelial carcinoma
CESC: cervical squamous cell carcinoma and endocervical adenocarcinoma
COAD: colon adenocarcinoma
KICH: kidney chromophobe
KIRC: kidney renal clear cell carcinoma
AML: acute myeloid leukemia
LUAD: lung adenocarcinoma
PCPG: pheochromocytoma and paraganglioma
PRAD: prostate adenocarcinoma
READ: rectum adenocarcinoma
SKCM: skin cutaneous melanoma
UCEC: uterine corpus endometrial carcinoma
UCS: uterine carcinosarcoma
ESAD: esophageal adenocarcinoma
MESO: mesothelioma
OSCC: oral squamous cell carcinoma
